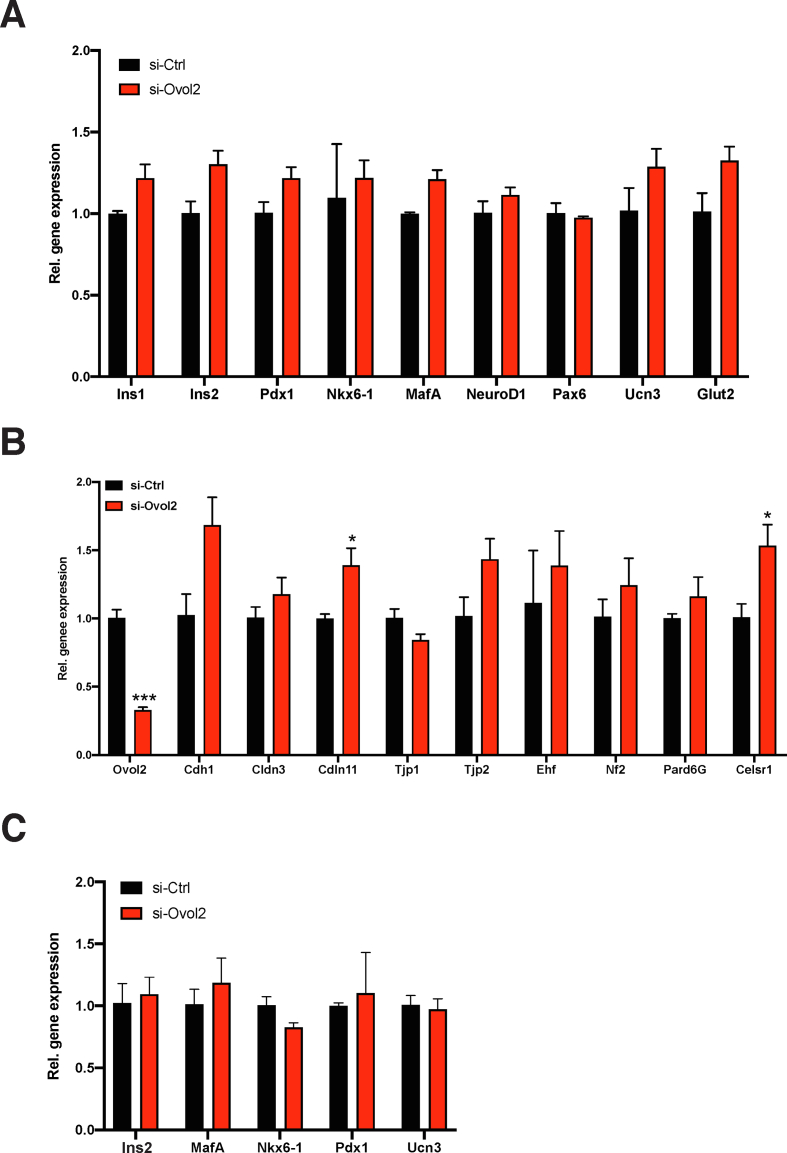# Dysregulation of the *Pdx1/Ovol2/Zeb2* axis in dedifferentiated β-cells triggers the induction of genes associated with epithelial–mesenchymal transition in diabetes

**DOI:** 10.1016/j.molmet.2021.101248

**Published:** 2021-05-12

**Authors:** Daniel S. de Jesus, Tracy C.S. Mak, Yi-Fang Wang, Yorrick von Ohlen, Ying Bai, Eva Kane, Pauline Chabosseau, Catherine M. Chahrour, Walter Distaso, Victoria Salem, Alejandra Tomas, Markus Stoffel, Guy A. Rutter, Mathieu Latreille

**Affiliations:** 1 Cellular Identity and Metabolism Group, MRC London Institute of Medical Sciences, Du Cane Road, London W12 0NN, UK; 2 Institute of Clinical Sciences (ICS), Faculty of Medicine, Imperial College London, Du Cane Road, London W12 0NN, UK; 3 Computing and Bioinformatics Facility, MRC London Institute of Medical Sciences, Du Cane Road, London W12 0NN, UK; 4 Imperial College Business School, London, UK; 5 Section of Cell Biology and Functional Genomics, Division of Diabetes, Endocrinology and Metabolism, Department of Metabolism, Digestion and Reproduction, Imperial College London, Du Cane Road, London W12 0NN, UK; 6 Institute of Molecular Health Sciences, ETH Zurich, Otto-Stern Weg 7, 8093 Zurich, Switzerland; 7 Lee Kong China School of Medicine, Nan Yang Technological University, Singapore

**Keywords:** Diabetes, Insulin, Pancreatic β-cells, Dedifferentiation, Epithelial-to-mesenchymal transition, microRNA, β-TFs, β-cell transcription factors, ECM, extracellular matrix, FACS, fluorescence-activated cell sorting, GSIS, glucose-induced stimulated-insulin secretion, GSEA, gene set enrichment analysis, microRNA, miRNA, scRNA-seq, single-cell RNA sequencing, T1D, type 1 diabetes, T2D, type2 diabetes, UPR, unfolded protein response

## Abstract

**Objective:**

β-cell dedifferentiation has been revealed as a pathological mechanism underlying pancreatic dysfunction in diabetes. We previously showed that increased miR-7 levels trigger β-cell dedifferentiation and diabetes. We used β-cell-specific miR-7 overexpressing mice (Tg7) to test the hypothesis that loss of β-cell identity triggered by miR-7 overexpression alters islet gene expression and islet microenvironment in diabetes.

**Methods:**

We performed bulk and single-cell RNA sequencing (RNA-seq) in islets obtained from β-cell-specific miR-7 overexpressing mice (Tg7). We carried out loss- and gain-of-function experiments in MIN6 and EndoC-bH1 cell lines. We analysed previously published mouse and human T2D data sets.

**Results:**

Bulk RNA-seq revealed that β-cell dedifferentiation is associated with the induction of genes associated with epithelial-to-mesenchymal transition (EMT) in prediabetic (2-week-old) and diabetic (12-week-old) Tg7 mice. Single-cell RNA-seq (scRNA-seq) indicated that this EMT signature is enriched specifically in β-cells. These molecular changes are associated with a weakening of β-cell: β-cell contacts, increased extracellular matrix (ECM) deposition, and TGFβ-dependent islet fibrosis. We found that the mesenchymal reprogramming of β-cells is explained in part by the downregulation of *Pdx1* and its inability to regulate a myriad of epithelial-specific genes expressed in β-cells. Notable among genes transactivated by *Pdx1* is *Ovol2*, which encodes a transcriptional repressor of the EMT transcription factor *Zeb2*. Following compromised β-cell identity, the reduction in *Pdx1* gene expression causes a decrease in *Ovol2* protein, triggering mesenchymal reprogramming of β-cells through the induction of *Zeb2*. We provided evidence that EMT signalling associated with the upregulation of *Zeb2* expression is a molecular feature of islets in T2D subjects.

**Conclusions:**

Our study indicates that miR-7-mediated β-cell dedifferentiation induces EMT signalling and a chronic response to tissue injury, which alters the islet microenvironment and predisposes to fibrosis. This research suggests that regulators of EMT signalling may represent novel therapeutic targets for treating β-cell dysfunction and fibrosis in T2D.

## Highlights

• Pancreatic β-cell dedifferentiation triggers an EMT gene programme in diabetes.
• Induction of TGFβ signalling in dedifferentiated β-cells alters islet microenvironments and contributes to islet fibrosis.
• *Pdx1* inhibits EMT signalling by transactivating *Ovol2*, a repressor of *Zeb2* in mature β-cells.
• Inverse correlation between *Pdx1* and *Zeb2* gene expression in islets from T2D subjects.

## 1 Introduction

Pancreatic β-cells are specialised cells within the islets of Langerhans enabling concerted secretion of insulin in response to elevated glycaemia [1,2]. Deficiency of functional insulin-producing β-cell mass leads to an increase in glycaemia and underlies the rising prevalence of diabetes worldwide (WHO 2019, https://www.who.int). Type 1 diabetes (T1D) is associated with autoimmune destruction of β-cells, whereas type 2 diabetes (T2D) is associated with the gradual deterioration of pancreatic β-cell function and survival induced by obesity and insulin resistance [3,4]. Impairment in glucose-stimulated insulin secretion (GSIS) has been attributed to changes in glucose sensing [5] and mitochondrial dysfunction [6], whereas prolonged endoplasmic reticulum and oxidative stress triggers β-cell apoptosis [7].

However, several studies have challenged our view of the prevalent pathogenic drivers of β-cell dysfunction in diabetes. β-cell dedifferentiation has been revealed as an alternative mechanism to β-cell apoptosis in T2D [8]. Dedifferentiated β-cells lose insulin expression and other markers of functional identity, including transcription factors and regulators of GSIS. High metabolic demand during pregnancy [8], insulin resistance [9,10], inflammation [8,11], and viral infection [12] have been shown to trigger β-cell dedifferentiation. Loss-of-function studies in mice have revealed that several β-cell transcription factors (β-TFs) (for example, *Pdx1*, *Nkx6-1*, *Nkx2.2*, *Pax6*, and *Rfx6*) [[13], [14], [15], [16], [17]] and epigenetic regulators (for example, *Dnmt1*, *Limd1*, and *Eed*/*Prc2*) [[18], [19], [20]] driving β-cell lineage development during embryogenesis are required to maintain the identity of mature β-cells. Furthermore, dysregulation of microRNA (miRNA) expression has been shown to impact β-cell identity [21]. Genetic deletion of miR-7 in β-cells results in improved β-cell function and glucose tolerance in mice through upregulation of several regulators of vesicle exocytosis and SNARE activity, including α-SYNUCLEIN (SNCA) and DNAJC5 (also referred to as CSPA) [22]. Moreover, we and others have reported that *miR-7* levels are increased in islets of *db/db* mice and non-obese Goto Kakizaki rats as well as in human islets transplanted into mice exposed to an obesogenic diet [22,23]. The pathological significance of these observations was demonstrated following the generation of β-cell-specific miR-7 overexpressing mice (Tg7) that develop early onset diabetes associated with a striking downregulation of transcripts encoding insulin and other β-cell differentiation markers including *Pax6*, a direct target of miR-7 [22,24], as well as *Pdx1*, *Mafa*, and *Slc2a*2 (herein referred to as G*lut2*). Furthermore, the expression of *Sox9*, a pancreatic progenitor marker, is specifically reactivated in mutant Tg7 islets [22,25]. However, unlike *db/db* mice, Tg7 mice do not develop obesity, making them a suitable model to study how β-cell dedifferentiation, independently of obesity, impacts gene expression and islet microenvironment.

Epithelial-mesenchymal transition (EMT) is a dynamic process playing a crucial role during embryonic development and in tissue repair and regeneration. Conversely, chronic activation of EMT signalling triggers mesenchymal reprogramming of different cell types and contributes to the development of several diseases, including organ fibrosis and cancer [26]. Previous studies revealed that a subset of EMT markers is expressed in both α- and β-cells of individuals with T2D [27,28]. Why and how EMT signalling is induced in T2D islets remain unknown. Herein, using Tg7 mice, a model of β-cell dedifferentiation, and diabetes, we report that loss of β-cell identity induces EMT-related genes. We found that this EMT process triggers a change in β-cell morphology and islet cell:cell contacts, leading to extracellular matrix deposition and fibrosis. At the molecular level, we uncovered that this EMT signature results in part from the inability of β-TFs to transactivate a subset of epithelial-specific genes and repressors of EMT signalling. More specifically, we discovered that *Pdx1* prevents mesenchymal reprogramming of β-cells by maintaining the expression of *Ovol2*, an epithelial-specific repressor of *Zeb2*. Together, our findings indicate that miR-7-mediated β-cell dedifferentiation triggers EMT signalling and a process reminiscent of a response to tissue injury predisposing to islet fibrosis in diabetes.

## 2 Materials and methods

### 2.1 Mouse lines and housing conditions

All the animals were kept and bred in a pathogen-free animal facility according to the Home Office regulations as defined by the Animal (Scientific Procedures) Act of 1986 and complied to ARRIVE guidelines. The mice were housed in Allentown XJ individually ventilated cages (IVCs) on a 12-h light/dark cycle with constant environmental conditions (temperature: 21 °C ± 2 °C, humidity: 55% ± 10%) and had free access to standard rodent chow. All the procedures were performed in accordance with UK Home Office regulations under HO Project Licence number 70/8967 (M. Latreille). Tg7 mice were previously described [22] and maintained by breeding males with C57BL/6J females. Mutant mice bearing rat *Ins2*-*Cre* [29] and *Rosa26*-floxed-Stop-*tdTomato* transgenes [30] (both in C57BL/6J backgrounds) were used to genetically label β-cells with a *tdTomato* fluorescence reporter. Blood glucose levels were measured by tail venesection. Male mice were used in all experiments.

### 2.2 Pancreatic islet isolation

Pancreatic islets were isolated following perfusion of the pancreas via the bile duct with 0.2 mg/ml of ice-cold Liberase (Roche) as previously described [22]. For dissociation studies, up to 600 islets were placed into 1.5 ml microtubes and washed three times with PBS. The islets were then incubated with Accutase at 37 °C for 8 min, and the reaction was stopped by adding foetal bovine serum (FBS). Islet cells were centrifuged at 1,200 RPM for 3 min and resuspended in RPMI 1640 medium, 11 mM of glucose supplemented with 10% FBS, 2 mM of l-glutamine, 100 IU/ml of penicillin, and 100 ug/ml of streptomycin and seeded in 24-well plates on coverslips coated with conditioned media from 804G cells [31] at the maximum density of 200,000 cells per well. For collagen I measurements, islets from Wt and Tg7 mice (16–18 weeks of age) were isolated and treated with TGFβ receptor 1 Alk5i inhibitor for 8 days. The supernatant was collected, kept on ice, and processed using a Mouse Collagen Type I (COL1) ELISA kit (Abbexa, Cambridge, UK) according to the manufacturer's instructions.

### 2.3 Flow cytometry analysis and pancreatic β-cell purification by fluorescence-activated cell sorting (FACS)

Pancreatic islets from Wt and Tg7 mice were isolated, dissociated, stained for selected antibodies, and analysed by flow cytometry. Briefly, wild-type and Tg7 islets were isolated and dissociated as previously described, centrifuged at 1,200 RPM for 1 min, washed and resuspended with PBS, and placed in a 96-well clear round bottom plate (Corning Inc., Corning, NY, USA). The samples were centrifuged at 1,200 RPM for 3 min and fixed with 4% formaldehyde (Sigma–Aldrich) for 30 min on ice. Islet cells were then centrifuged at 1,200 RPM for 3 min, washed twice with 1x PBS, and permeabilised with permeabilisation buffer (1:10 in H_2_O) (eBioscience kit, Thermo Fisher Scientific, USA) for 15 min on ice. Primary and secondary antibodies were diluted in permeabilisation buffer (1:100 and 1:500, respectively) and added to islet cells for 1 h. The samples were then centrifuged at 1,200 RPM for 3 min, washed in PBS, resuspended in 150 μl of FACs buffer (2% FBS and 2 mM EDTA in 1x PBS), and transferred into a round-bottomed tube containing a cell strainer cap (Corning Inc., Corning, NY, USA). Intracellular protein staining was performed using a BD LSRII flow cytometer and analysed using FlowJo software (version 10). A minimum of 10,000 events were recorded for each sample. Doublet cells were identified and excluded and geometric mean fluorescence intensity was obtained and normalised by the total number of single cells. For fluorescence-activated cell sorting (FACS), islets from Wt and Tg7 mice with rat Ins2 (RIP)-Cre carrying a Rosa26-floxed-Stop-*tdTomato* transgene were isolated, dissociated, and washed twice with 1x PBS, centrifuged at 1,200 RPM for 3 min, resuspended in 1 ml of FACs buffer, and placed in a round-bottomed tube with a cell strainer cap (Corning Inc., Corning, NY, USA). *TdTomato*^+^ β-cells were isolated using a BD FACSAria III Cell Sorter and used for RNA extraction.

### 2.4 Histology and immunofluorescence

At least three sections ∼200 μm apart from three animals of each genotype were used for every analysis. The sections were stained with haematoxylin and eosin as previously described (Fischer et al., 2014). Pancreatic sections were stained using a Picro Sirius Red Stain kit as described by the manufacturer (Abcam). For immunofluorescence staining, pancreata were dissected, weighed, and fixed in 4% PFA at 4 °C overnight. The sections were deparaffinised and rinsed in distilled water for 5 min. Antigen retrieval was performed where necessary using a Decloaking Chamber NxGen (BioCare Medical, USA) for 5 min at 110 °C for 5 min in Tris–HCl at a pH 10.0 or sodium citrate at a pH of 6.0. The sections were permeabilised for 30 min in permeabilisation buffer (0.1% Triton/PBS) and blocked for 1 h at room temperature in blocking buffer (1% bovine serum albumin/5% serum/PBS). Primary antibodies were diluted in blocking buffer and incubated on sections overnight at 4 °C. The slides were washed three times for 15 min in permeabilisation buffer. The sections were then incubated with a fluorochrome-conjugated secondary antibody diluted in blocking buffer for 1 h at RT in the dark. The slides were rinsed and mounted on glass slides with VectaShield. Dissociated islets were fixed in 4% PFA/PBS for 15 min at room temperature followed by 2 washes with PBS. Islet cells were incubated with permeabilisation buffer for 15 min at room temperature followed by three washes with PBS and blocked in blocking buffer for 1 h at room temperature. Primary and secondary antibodies were added as previously described. Images were acquired using a Leica SP5II or an Olympus IX70 and analysed using ImageJ software.

### 2.5 Electron microscopy

For electron microscopy, islets were chemically fixed in 2% paraformaldehyde (EM grade), 2% glutaraldehyde, and 3 mM of CaCl_2_ in 0.1 M of cacodylate buffer for 2 h at room temperature, then left overnight at 4 °C in fresh fixative, osmicated, enrobed in agarose plugs, sequentially dehydrated in ethanol, and embedded on Epon polymerised overnight at 60 °C. Ultrathin 70 nm sections were cut with a diamond knife (DiATOME) using a Leica Ultracut UCT ultramicrotome before examination on a FEI Tecnai G2 Spirit TEM. Images were acquired in a charge-coupled device camera (Eagle) and processed with ImageJ. For calcium imaging, islets were loaded with fluo-8 and imaging was performed as previously described [[32], [33], [34]] using a Nipkow spinning disk head. In brief, a solid-state laser (CrystaLaser) controlled by a laser merge module (Spectral Applied Physics) provided wavelengths of 491 nm (rate, 0.5 Hz and exposure time, 600 ms). Emitted light was filtered at 525/50 nm, and images were captured with a 16-bit 512 × 512 pixels back-illuminated EM-CCD camera (ImageM 9100-13, Hamamatsu) driven by Volocity software (PerkinElmer Life Sciences). For connectivity and correlation analyses, individual β-cell regions of interest (ROIs) were visually identified within each dye-loaded isolated islet that was imaged (typically ∼50 per islet). Mean fluorescence intensity time plots from each ROI were subjected to correlation analyses for all cell pairs using a custom-made MATLAB script (available upon request). Data were smoothed using a retrospective averaging method (previous 10 values), and all traces were normalised to F0. The Pearson correlation function R between all possible (smoothed) cell pair combinations (excluding the autocorrelation) was assessed, and the data were subsequently subjected to bootstrap resampling to increase the accuracy of the confidence interval of the R statistic, with p < 0.001 deemed a statistically significant cell–cell connection. Connectivity data were displayed in two formats. First, the Cartesian coordinates of the imaged cells within a given islet were used to construct connectivity line maps. Cell pairs (R > 0.25 and p < 0.001 post bootstrap) were connected with a straight line, the colour of which represented the correlation strength (R = 0.25–0.5 [green], R = 0.5–0.75 [yellow], and R = 0.75–1.0 [red]). Second, Pearson heatmap matrices indicating individual cell pair connections on each axis (min. = −1; max. = 1) were produced.

### 2.6 Cell culture, transfection, and viral infections

MIN6 cells were cultured in Dulbecco's Modified Eagle Medium with 4.5 g/L of glucose and phenol red (DMEM, Thermo Fisher Scientific, UK) supplemented with 15% FBS (Eurobio), 1% penicillin-streptomycin (Thermo Fisher Scientific), 1% GlutaMAX (Thermo Fisher Scientific), 100 mM of 1% sodium pyruvate (Thermo Fisher Scientific), and 0.0005% β-mercaptoethanol (Sigma–Aldrich, UK). HEK293T cells were cultured in DMEM with 5% FBS, 1% penicillin-streptomycin, and 1% GlutaMAX. The cells were transfected with siRNA (Horizon Discovery) using DharmaFECT1 as recommended by the manufacturer. Adenovirus infections were performed as previously described [22]. For luciferase assays, HEK293 cells were plated at the density of 5 × 10^4^ cells/well in a 24-well plate and co-transfected with 50 ng of pGL3 Basic Firefly luciferase reporter plasmids and increasing amounts of V5-tagged *Pdx1* or *tdTomato* control and 25 ng of pRL-TK Renilla Firefly reporter vectors using Lipofectamine 2000 (Thermo Fisher Scientific). After 48 h, the luciferase activity was measured using a Dual-Luciferase Reporter Assay System (Promega) on a Centro LB960 luminometer (Berthold Technologies). The ratio between the Firefly/Renilla luciferase activity was then calculated.

### 2.7 Plasmid DNA constructions

A fragment containing an evolutionary conserved *Pdx1*-binding site within *Ovol2* Intron 3 was amplified by PCR using Phusion High-Fidelity DNA polymerase (Thermo Fisher Scientific) from C57BL/6J mouse genomic DNA. The amplified DNA fragment was cloned at *XhoI* and *HindIII* sites of pGL3 luciferase reporter vector (Promega). The *Pdx1* coding sequence was amplified by PCR using Phusion High-Fidelity DNA polymerase from MIN6 cDNA and cloned into a pcDNA 3.1/V5-His TOPO vector (Thermo Fisher Scientific) following the manufacturer's instructions. Clones were verified by Sanger sequencing.

### 2.8 RNA extraction, quantitative PCR, and western blotting

RNA was extracted using TRIzol and treated with DNaseI using RNAse-free DNAse sets (Thermo Fisher Scientific) based on the manufacturer's recommendations. Reverse transcription was performed using a High-Capacity cDNA Reverse Transcription kit (Thermo Fisher Scientific) and quantitative PCR was performed using a KAPA SYBR FAST qPCR Master Mix kit (Kapa Biosystems) on a LightCycler 480 apparatus (Roche). Data were normalised over the housekeeping gene *RPLP0*. For western blotting, cells lysed in RIPA buffer containing 20 mM TRIS–HCl pH 7.5, 150 mM NaCl, 1 mM EDTA 1% NP-40, 1% sodium deoxycholate, and supplemented with proteinase inhibitor (Roche).

### 2.9 Bulk RNA-sequencing analysis

RNA was extracted from islets of 2-week-old and 12-week-old Tg7 and littermate Wt controls (pools from at least 3 animals). RNA-seq libraries were prepared with an NEB Ultra II RNA library kit (Illumina) from 10 ng total. Sequencing then proceeded with Hiseq2500 using paired-end 100 bp reads at the MRC LMS Genomics facility. Illumina CASAVA 1.8.4 software was used for base calling and demultiplexing. Raw RNA-seq reads were trimmed with trimmomatic to remove adaptors and low-quality reads (v.0.33) [35] and then aligned against the Ensembl *Mus musculus* genome reference sequence assembly (mm9) and transcript annotations using TopHat2 (2.0.11) [36]. Gene-based read counts were then obtained using the featureCounts function [37] from the Rsubread Bioconductor package [38]. Differential expression analysis was performed using the DESeq2 Bioconductor package [39] and an adjusted p value was obtained using the Benjamini-Hochberg method. A ranked gene list based on Wald statistics from DESeq2 results was used with GSEA with MSigDB gene sets from H collections [40,41]. The epithelial genes list comprised genes with a function in epithelial cells based on the UniProt database, whereas a mesenchymal-related gene list was generated from the dbEMT gene set (http://dbemt.bioinfo-minzhao.org/), both of which can be found in Supplementary Table 6. RNA-seq data sets from *db/db* and T2D individuals were obtained from [42,43], respectively.

### 2.10 Single-cell RNA-sequencing analysis

Islets were isolated from 12-week-old Tg7 and littermate Wt mice, dissociated into single-cell suspensions, and used for library preparation (10x Genomics) at the MRC LMS Genomic facility. cRNA-seq data were demultiplexed and aligned against the mouse mm10 genome with CellRanger v3.0.2 [44] and bcl2fastq v2.17.1.14. The R bioconductor package Seurat 3.1.1 [45,46] was applied for the subsequent analysis. After removing doublets with DoubletsFinder [47], scRNA-seq from the Wt and Tg7 mice was normalised using the SCTransform [48] function with the argument “vars.to.regress = percent.mt” before integrating using FindIntegrationAnchors and IntegrateData functions from Seurat. Processed data were then visualised by UMAP using the RunUMAP function from Seurat.

### 2.11 CUT&Tag

CUT&Tag experiments were carried out using a protocol developed by Kaya-Okur et al. [49]. Briefly, 1 × 10^6^ MIN6 cells were washed twice with 1.5 ml of wash buffer (20 mM of HEPES at a pH 7.5, 150 mM of NaCl, 0.5 mM of spermidine, and 1 × protease inhibitor cocktail from Roche) and centrifuged at 600 g for 3 min. Concanavalin A-coated magnetic beads (Bangs Laboratories) were washed twice with 1.5 ml of binding buffer (20 mM of HEPES at a pH 7.5, 10 mM of KCl, 1 mM of CaCl_2_, and 1 mM of MnCl_2_) and 10 μl was added to each cell preparation and incubated on an end-over-end rotator for 10 min at room temperature. The beads were resuspended in 50 μl of DIG Wash buffer (20 mM of HEPES at a pH 7.5, 150 mM of NaCl, 0.5 mM of spermidine, 0.05% digitonin, 2 mM EDTA, 30% BSA, and 1 × protease inhibitor cocktail) containing 1 μg of primary antibody. The samples were incubated on a nutator for 2 h at room temperature. Then 2 μl of secondary antibody guinea pig anti-rabbit IgG was diluted in 100 μl of DIG Wash buffer was added to the samples and incubated for 30 min at room temperature. The samples were washed twice with 800 μl of DIG Wash buffer for 5 min. A pA–Tn5 enzyme/adapter complex was diluted to 1:200 in Dig-300 buffer (0.05% digitonin, 20 mM of HEPES pH of 7.5, 300 mM of NaCl, 0.5 mM of spermidine, and 1 × protease inhibitor cocktail) and 100 μl was added to the samples and incubated for 1 h at room temperature. The samples were washed twice with 800 μl of Dig-300 buffer for 5 min and resuspended in 300 μl of tagmentation buffer (10 mM of MgCl_2_ in Dig-300 buffer) and incubated for 1 h at 37 °C. Tagmentation was terminated by adding 10 μl of 0.5 M EDTA, 3 μl of 10% SDS, and 2.5 μl of 20 mg/ml proteinase K, and the samples were incubated for 1 h at 55 °C. Then 300 μl of phenol-chloroform-isoamyl alcohol 25:24:1 (Thermo Fisher Scientific) was added to each sample, mixed, and centrifuged at 16,000 *g* for 3 min. Chloroform was added to each sample and centrifuged at 16,000 *g* for 3 min. DNA was then precipitated with 100% ethanol and centrifuged at 16,000 *g* for 15 min at 4 °C. The pellets were rinsed with 1 ml of ethanol and centrifuged for 1 min at 4 °C (16,000 g), air-dried, and resuspended in 30 μl of 10 mM Tris–HCl at a pH 8.0, 1 mM EDTA, and 25 ug/ml of RNAse A and incubated for 10 min at 37 °C. Next, 33 μl of Ampure XP beads (Beckman Counter) were added to each tube, quickly spun, and incubated for 10 min at room temperature. The beads were then washed twice with 80% ethanol and air-dried for 5 min. Then 25 μl of 10 mM Tris at a pH 8.0 was added to each sample, full-speed vortexed for 2 s, and placed on a magnetic stand. Liquid containing DNA was pipetted, and the DNA libraries were prepared using a NEBNext Library kit (Illumina). Post-PCR clean-up was performed by adding 1.1 volume of Ampure XP beads to each sample and incubating for 15 min at room temperature. The beads were washed twice with 80% ethanol. DNA fragments were eluted with 30 μl of 10 mM Tris at a pH 8.0 and used for quantitative PCR.

### 2.12 Statistical analysis

Data are reported as mean ± standard error of the mean (SEM). Statistical significance was obtained using Student's t-test or ANOVA with Sidak's post hoc tests using Prism 7 (GraphPad software). P < 0.05 was considered significant.

## 3 Results

### 3.1 Loss of insulin expression and altered β-cell homeostasis in Tg7 mice

Mice overexpressing miR-7 specifically in pancreatic β-cells (∼3-fold) develop diabetes due to β-cell dedifferentiation [22]. In the current study, we used Tg7 mice to investigate how the loss of β-cell identity impacts the islet microenvironment. First, we performed microscopic analyses on pancreatic sections from normoglycaemic 2-week-old (2w) and hyperglycaemic 12-week-old (12w) Tg7 mice (Figure 1A). Immunohistochemistry revealed a subtle difference in insulin content between the Wt and Tg7 islets at 2w of age despite mutant mice maintaining normal blood glucose and circulating insulin concentrations (Figure 1B). In contrast, islets from the 12w Tg7 mice showed a marked decrease in insulin signals compared to controls. To confirm these findings, we performed flow cytometry using islet cells from Tg7 mice bearing an insulin-*Cre*-driven *Rosa26 tdTomato* reporter (Wt^β−Tom^ and Tg7^β−Tom^ mice). This evaluation indicated that most *tdTomato*-labelled β-cells from the Tg7 mice displayed a low degree of granularity (lower side scatter, SSC) than β-cells from the Wt mice (Figure 1C). Electron microscopy in islets from the 12w Tg7 mice confirmed these findings by revealing a more significant number of cells utterly devoid of electron-dense insulin granules, so-called “empty” cells, as well as the presence of β-cells with rod-shaped dense cores (Figure 1D and Supplementary Figure 1). This degranulation of β-cells correlated with profound alterations of β-cell homeostasis as indicated by impaired Ca^2+^ influx in response to high glucose compared to controls (Supplementary Figure 2). Together, these analyses revealed that β-cells from the Tg7 mice underwent a progressive degranulation process that correlated with altered β-cell homeostasis and diabetes.

**Figure 1:**
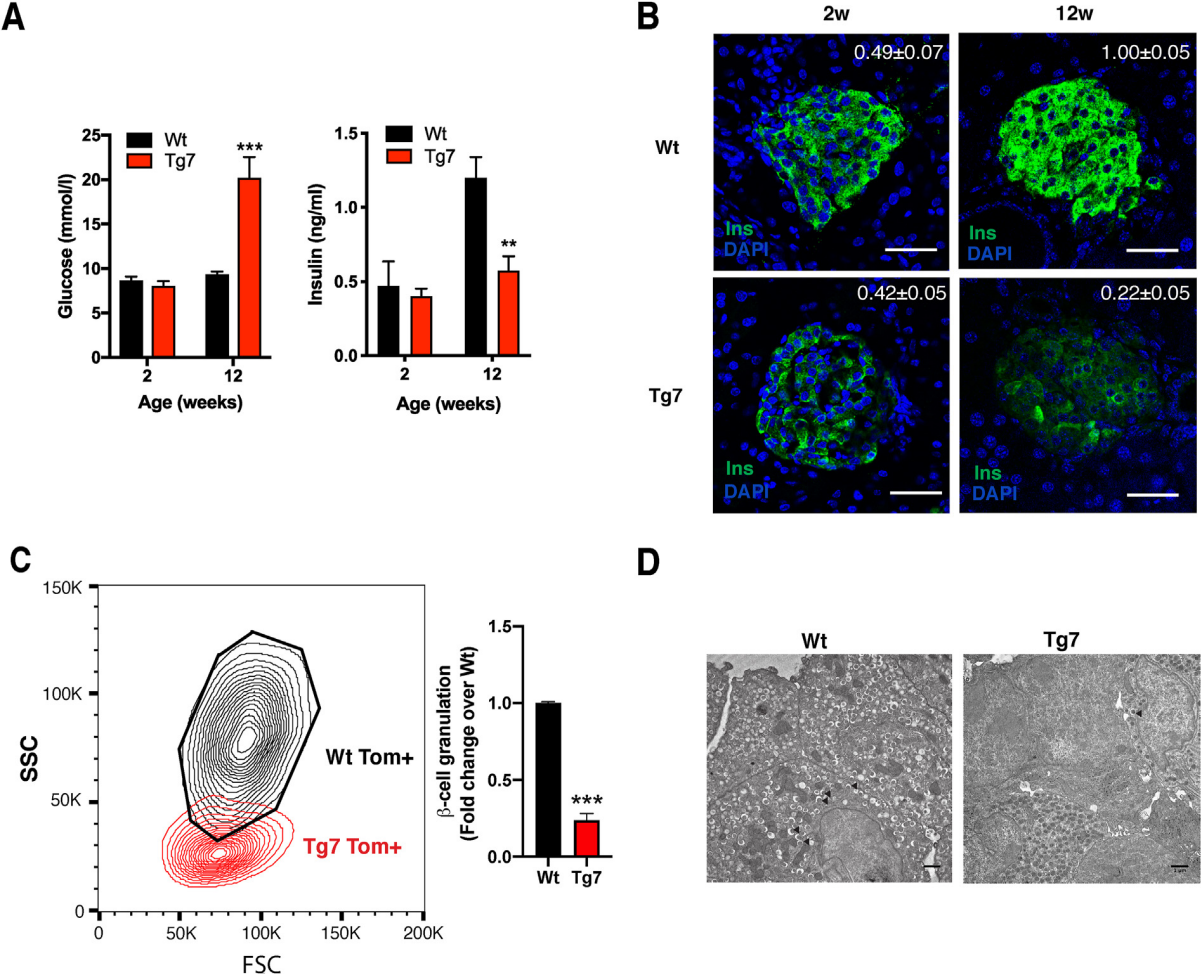
**Pancreatic β-cell degranulation and dysfunction in Tg7 mice**. **(**A) Random fed glycaemia and circulating insulin levels in 2- and 12-week-old Wt and Tg7 mice. (B) Insulin (green) immunofluorescence of pancreatic sections from wild-type (Wt) and Tg7 mice at 2w and 12w of age. Nuclei revealed by DAPI staining. Average insulin immunostaining quantification (n = 4–5). Scale bar: 75 μm. P < 0.001 12w Tg7 vs 12w Wt; p < 0.05 12w Tg7 vs 2w Tg7. (C) Flow cytometry in dissociated islets from 12w Wt^β−Tom^ and Tg7^β−Tom^ mice showed the presence of β-cells with decreased granulation in mutant mice. Right: Quantification of degranulated Tom^+^ β-cells from Tg7 mice residing outside the Wt gate (black area considered normal β-cell granularity) expressed as fold change over Wt mice (n = 5). (D) Electron microscopy of 12w Wt and Tg7 islet preparations. Black arrowheads: mature insulin granules. Scale bar: 1 μm. Unpaired Student's t-test. Data are means ± SEM, ∗∗p < 0.01 and ∗∗∗p < 0.001.

### 3.2 Genetic signature of islets from Tg7 mice

To clarify how β-cell dedifferentiation induced by miR-7 impacts whole islet gene expression, we performed bulk RNA-seq analyses in Tg7 islets isolated at 2w and 12w. A total of 2,471 genes were differentially regulated in the 2w islets (211 genes downregulated and 735 upregulated with at least a 2-fold change [2-FC], padj < 0.05), whereas this reached 4,220 in the 12w Tg7 islets (348 downregulated and 1,110 upregulated by at least 2-FC, padj < 0.05) Of note, the expression of more than one-third of differentially expressed genes (1,457/4,220) in islets from the 12w hyperglycaemic Tg7 islets were already similarly altered in 2-week-old prediabetic Tg7 islets (Supplementary Table 1), revealing a pronounced remodelling of gene expression before the onset of diabetes. Gene set enrichment analysis (GSEA) [41] indicated that genes downregulated in 2w and 12w Tg7 islets displayed an overrepresentation of core β-cell components, including the unfolded protein response (UPR), pancreatic β-cell identity, and protein secretion (Figure 2A and Supplementary Table 2). Consistent with β-cell dedifferentiation in Tg7 mice, we found decreased expression of several β-cell-specific markers (Figure 2C) as well as increased expression of progenitor (*Sox9* and *Ngn3*) and stemness markers (Figure 2D) by quantitative RT-PCR (qPCR).

**Figure 2:**
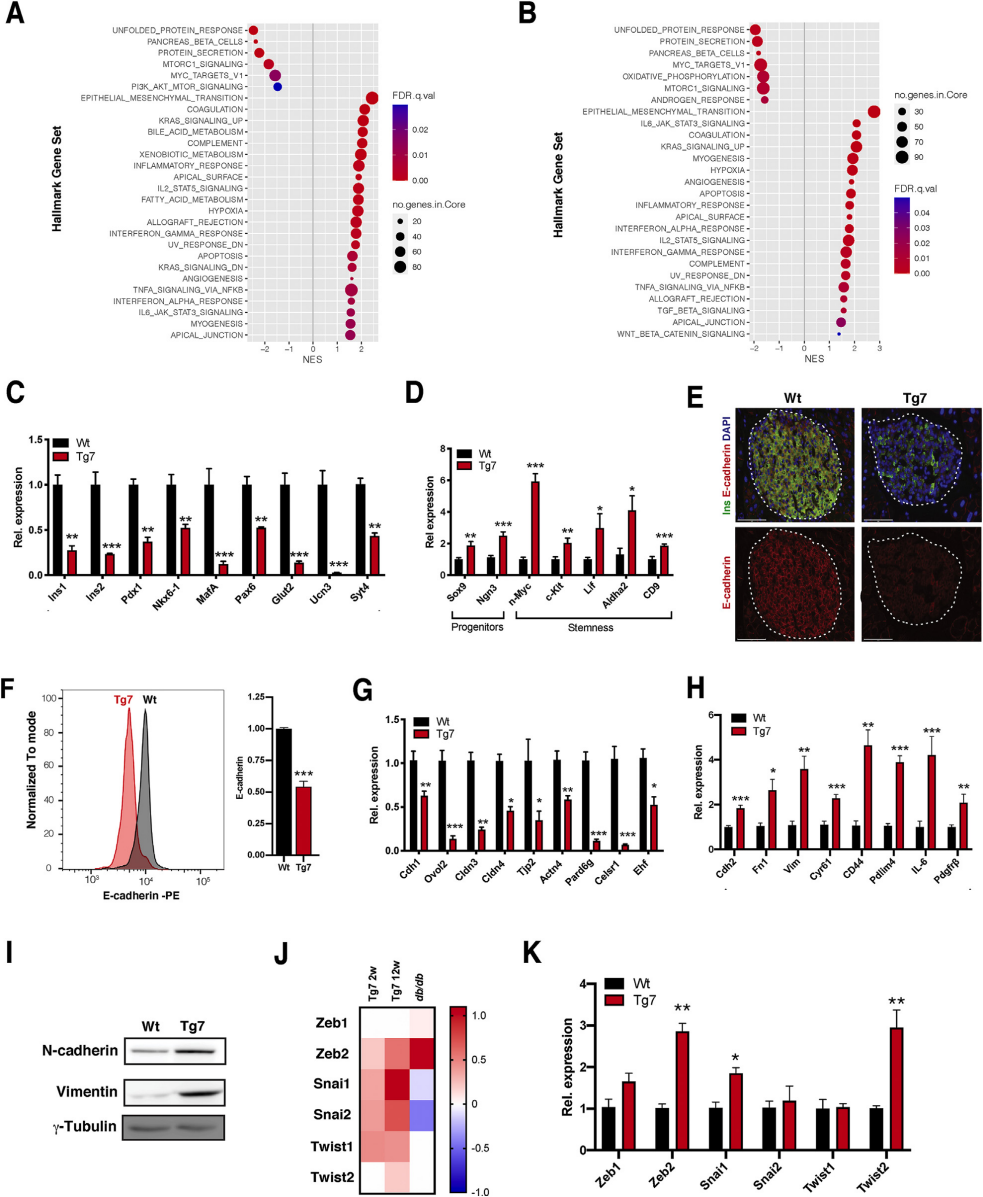
**β-cell dedifferentiation is associated with the induction of genes involved in EMT in Tg7 mice.** (A–B) Gene set enrichment analysis (GSEA) of MSigDB Hallmark genes in downregulated (A) and upregulated (B) pre-ranked gene ratios (Tg:Wt) from 2w (left) and 12w (right) Tg7 islets. Gene sets with the indicated FDR are plotted relative to normalised enrichment scores (NES). Circle size denotes the number of genes in each category, and colour indicates FDR q values. (C–D) qPCR analysis of β-cell identity genes (C) progenitor and stemness genes (D) in isolated islets from 12w mice (n = 6–7). (E) *E-cadherin* immunofluorescence in pancreas of 12w Tg7 and control Wt mice. Scale bar: 75 μm. (F) E-*cadherin* flow cytometry in dissociated islets from 12w Wt^β−Tom^ and Tg7^β−Tom^ mice. Data represent *tdTomato*^+^ -gated β-cells (n = 3). (G–H) qPCR analysis of epithelial (G) and mesenchymal markers (H) in islets isolated from 12w old mice (n = 6–7). (I) Western blotting for *N-cadherin* and vimentin in islets from 12w Tg7 mice. (J) Heatmap of EMT-TF expression data (log_2_ fold change) in islets from 12w Tg7 and *db/db* mice over their respective Wt controls. (K) qPCR analysis of EMT-TFs in islets from 12w Tg7 mice. Unpaired Student's t-test. Data are means ± SEM, ∗p < 0.05,∗∗p < 0.01, and ∗∗∗p < 0.001.

Interestingly, we found that the top category of upregulated genes was associated with epithelial-to-mesenchymal transition, TGFβ signalling, inflammation, angiogenesis, cell polarity, and myofibroblast activation (Figure 2B and Supplementary Table 2). These genetic signatures were, in large part, already an intrinsic feature of normoglycemic 2w Tg7 mice and further overrepresented in diabetic 12w Tg7 mice. Intriguingly, EMT was also the top biological process enriched in islets isolated from *db/db* mice (Supplementary Figure 2), indicating that EMT represents a core characteristic of different mouse models of diabetes. EMT signalling drives mesenchymal reprogramming of epithelial cells and underlies tumour metastasis in cancer [50]. Conversely, EMT plays a fundamental role in response to cellular dedifferentiation in non-epitheial tissues and contributes to tissue repair, plasticity, and regeneration [51]. Although islet endocrine cells express several epithelial-specific markers, including *Cdh1/E-cadherin* [52,53], they showed limited morphological and functional similarities to epithelial cells. This raises the intriguing possibility that loss of β-cell identity in T2D elicits an EMT process reminiscent of a response to tissue injury. To investigate this, we first measured the expression of *Cdh1/E-cadherin,* a marker downregulated during EMT. Immunofluorescence and flow cytometry analyses revealed a downregulation of *E-cadherin* protein expression in islets from diabetic Tg7 mice (Figure 2E, F). We found that several epithelial cell markers (*Ovol2*, *Cldn3/4*, *Tjp2*, and *Ehf*) and regulator of cell polarity (*Celsr1* and *Pard6g*) expressed in pancreatic β-cells were markedly downregulated in islets from diabetic Tg7 mice and *db/db* mice (Figure 2G and Supplementary Table 3). Conversely, Tg7 islets showed increased expression of core EMT markers such as *Cdh2/N-cadherin* and *Vim* (Figure 2H, I and Supplementary Table 3). We found that this EMT signature was a component of Tg7 and a feature of islets isolated from diabetic *db/db* islets (Supplementary Figure 3A), a mouse model displaying similar induction of miR-7 expression [22]. Given that the EMT GSEA signature is an indicator of the transcriptional response to EMT, we then verified the expression of EMT-TFs from the *Zeb*, *Snail*, and *Twist* gene families, which are key executioners of EMT. We found that *Zeb2* was the only EMT-TF consistently regulated in the diabetic Tg7 and *db/db* mice (Figure 2J, K and Supplementary Figure 3B, C). Together, our results indicated that loss of β-cell identity concurred with established changes in gene expression associated with EMT and a chronic response to tissue injury.

### 3.3 β-cell dedifferentiation in Tg7 mice is associated with changes in cell:cell contacts and islet fibrosis

TGFβ signalling is a crucial regulator of EMT and tissue fibrosis [50]. Because our bulk RNA-seq GSEA analysis identified a TGFβ signature in the Tg7 mice, we monitored TGFβ signalling activation by Western blotting using an antibody against phosphorylated SMAD3 on Ser 423/425 (p-SMAD3). These analyses revealed increased p-SMAD3 levels in islets from 12w Tg7 mice (Figure 3A). Moreover, this correlated with the induction of TGFβ ligands (Supplementary Table 4) and several TGFβ target genes in mutant islets compared to controls (Figure 3B). Given that TGFβ signalling induces morphological changes and marked alterations in tissue microenvironments following organ injury [54], we investigated islet β-cell morphology and β-cell:β-cell interactions in islets from Tg7^β−Tom^ mice. Our microscopic observations indicated that *tdTomato*^+^ β-cells from Tg7 mice changed shape from cuboidal to flattened and elongated and displayed loosened cell:cell contacts (Figure 3C), two features of TGFβ-induced EMT cell reprogramming. Electron microscopy confirmed the presence of a large cellular interspace, with some islet cells displaying well-defined protrusions (Figure 3D). We also found increased expression of several TGFβ-regulated extracellular matrix (ECM) remodellers in mutant islets, which correlated with enhanced collagen deposition in islets from diabetic Tg7 mice (Figure 3E, F). In particular, collagen I content was higher in Tg7 islets compared to Wt controls. Significantly, collagen I deposition was completely normalised following incubation with Alk5i, an inhibitor of TGFβ receptor type I and inhibitor of EMT (Figure 3G). Altogether, these results indicated that EMT and TGFβ signalling contributed to islet fibrosis in diabetic Tg7 mice.

**Figure 3:**
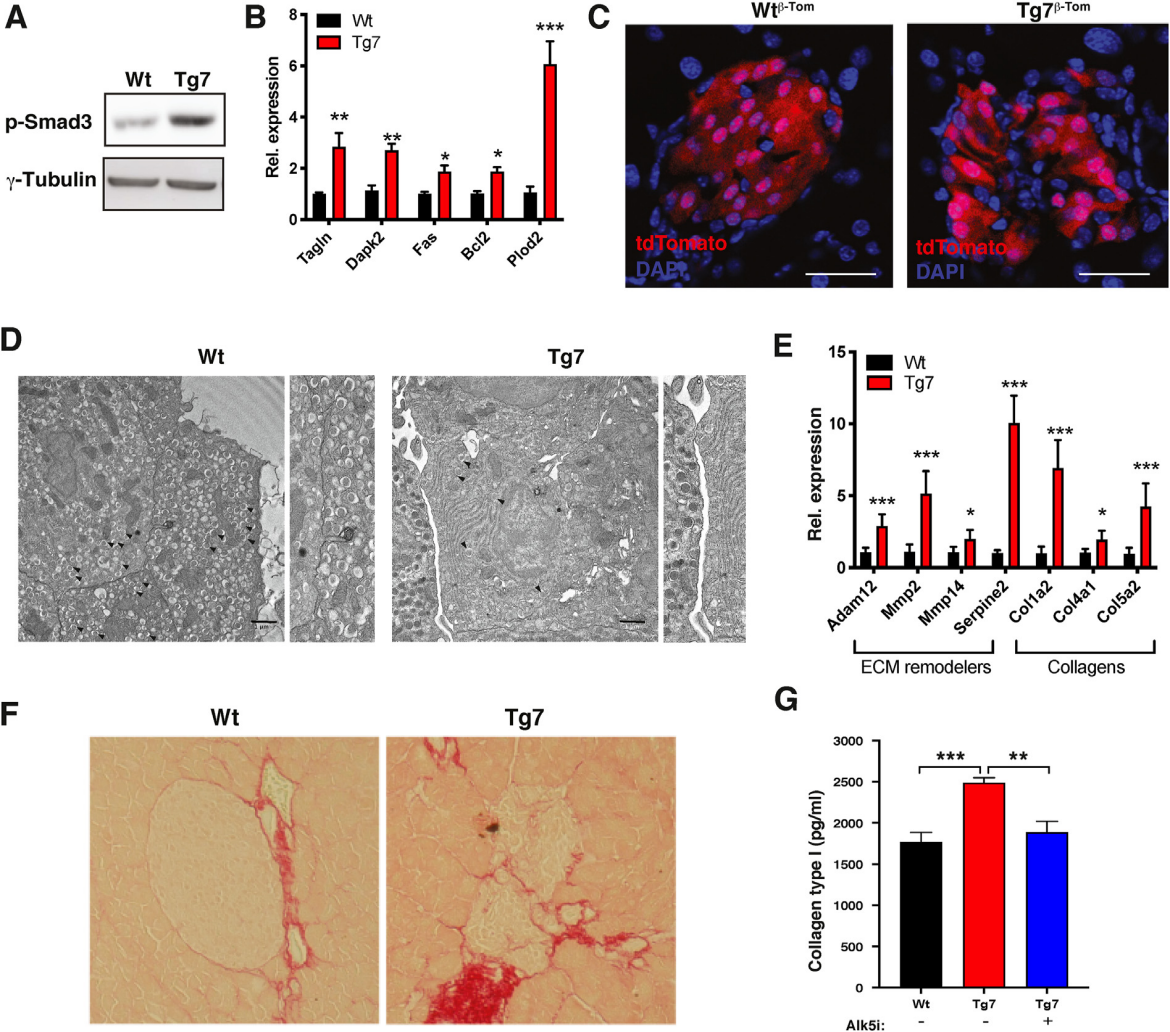
**Loss of β-cell identity is associated with alteration of islet microenvironment and TGFβ-dependent fibrosis**. (A) Western blotting for phospho-Ser423/425 and γ-tubulin in islets from 12w Tg7 mice. (B) mRNA levels for TGFβ target genes in islets from 12w Tg7 and Wt mice obtained by qPCR (n = 6). (C) Fluorescence microscopy on pancreatic sections from Wt^β−Tom^ and Tg7^β−Tom^ mice Red: *tdTomato* autofluorescence associated with β-cells. Scale bar: 40 μm. (D) Electron microscopy of 12w Wt and Tg7 islet preparations. Arrowhead indicates insulin granules. Arrows indicate the intercellular space between surrounding β-cells in Tg7 compared to control islets. Scale bar: 1 μm. Also shown is a zoom of the intercellular space between islet β-cells. (E) mRNA levels for ECM remodellers and collagen genes in islets from 12w Tg7 and Wt mice obtained by qPCR (n = 6). (F) Sirius red staining of pancreatic sections from 12w Tg7 mice and controls. (G) Collagen I content in islets from 12w Tg7 and Wt mice measured by ELISA. Islets from Tg7 mice were pre-treated with TGFβRI inhibitor Alk5i (1 uM) (n = 3–6). In panel G, one-way ANOVA with Sidak's multiple comparison test; otherwise, unpaired Student's t-test. Data are means ± SEM. ∗p < 0.05, ∗∗p < 0.01, and ∗∗∗p < 0.001.

### 3.4 Single-cell RNA-seq reveals induction of EMT gene specifically in β-cells of Tg7 mice

To define the contribution of islet cells to this EMT process, we performed single-cell RNA sequencing (scRNA-seq) in islets from 12w Wt^β−Tom^ and Tg7^β−Tom^ mice. After removing potential doublet cells, coarse clustering of gene expression identified different groups of cells corresponding to insulin-, glucagon-, and somatostatin-producing β-, α-, and δ-cells, respectively, whereas *Cd68* and *Pecam1* mRNA expression identified small clusters of tissue-resident macrophage and endothelial cells, respectively (Figure 4A, B). We also found that a cluster appeared to co-express *Ins2* and *Gcg* or *Sst* (poly cluster), and its proportion increased by 3-fold in the Tg7 mice (Supplementary Table 5). As expected, we found that several β-cell-specific and epithelial markers were downregulated in β-cells of the mutant mice, which correlated with the upregulation of several EMT-related genes (Figure 4C, D). In fact, the dysregulation of EMT-related genes was essentially observed only in β-cells, with a modest change in other endocrine cell types (Supplementary Figure 4A). To validate these findings, we isolated *tdTomato*-labelled β-cells from the Wt^β−Tom^ and Tg7^β−Tom^ mice by FACS and analysed them by qPCR (Figure 4E, F). As expected, *tdTomato*^+^ β-cells from the Tg7 mice displayed decreased expression of epithelial-specific genes including *Ovol2* (Figure 4G and Supplementary Figure 4B), whereas mesenchymal genes such as *Zeb2* increased specifically in dedifferentiated *tdTomato*^+^ β-cells from the Tg7 mice (Figure 4H and Supplementary Figure 4C). From these analyses, we concluded that genes involved in EMT were induced specifically in dedifferentiated β-cells from Tg7 mice.

**Figure 4:**
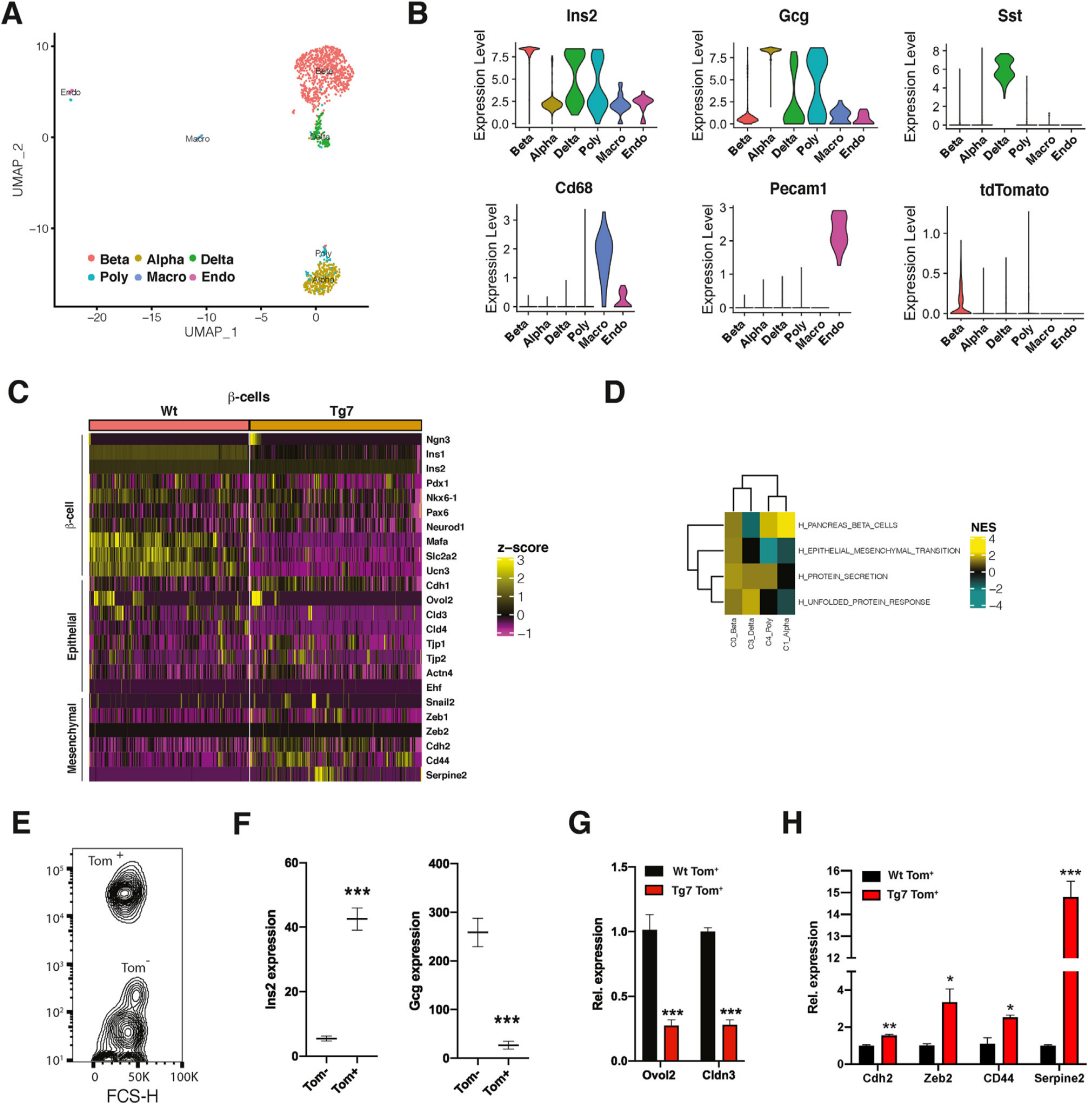
**scRNA-seq uncovers an EMT gene signature within β-cells of diabetic Tg7 mice**. (A) Uniform manifold approximation and projection (UMAP) visualisation of islets cells from 12w Wt and Tg7 mice as a merged dataset with each dot consisting of a single cell. (B) Expression patterns of endocrine cell markers *lns2*, *Gcg*, and *Sst* defining α-, β-, and δ-cells, respectively. Polyhormonal (Poly) cells are defined as cells displaying co-expressing *Ins2*, *Gcg*, and *Sst*. Macrophage (Macro) and endothelial (Endo) cells are defined by the expression of *Cd68* and *Peacam1*, respectively (C) Heatmap displaying differentially expressed genes in endocrine cell clusters from Wt and Tg7 mice. Magenta is low, black is intermediate, and yellow is high expression. (D) GSEA analysis of differentially expressed genes in endocrine islet cells of Tg7 mice. A selection of Hallmark gene sets is presented; FDR <0.25 (E) Contour plot of FACS-sorted *tdTomato*^+^ (Tom^+^) and *tdTomato*^*-*^ (Tom^−^) cells from dissociated islets of Wt^β−Tom^ mice (F) Expression of *Ins2* and *Gcg* in Tom^+^ and Tom^−^ cells analysed by qPCR. Data related to data presented in panel E. (G) Expression of epithelial and mesenchymal markers in Wt Tom^+^ and Tg7 Tom^+^ cells obtained by FACS analysed by qPCR (n = 3). Unpaired Student's t-test. Data are means ± SEM. ∗p < 0.05, ∗∗p < 0.01, and ∗∗∗p < 0.001.

### 3.5 Cell-autonomous β-cell upregulation of EMT genes in response to elevated miR-7 levels

**Figure 5:**
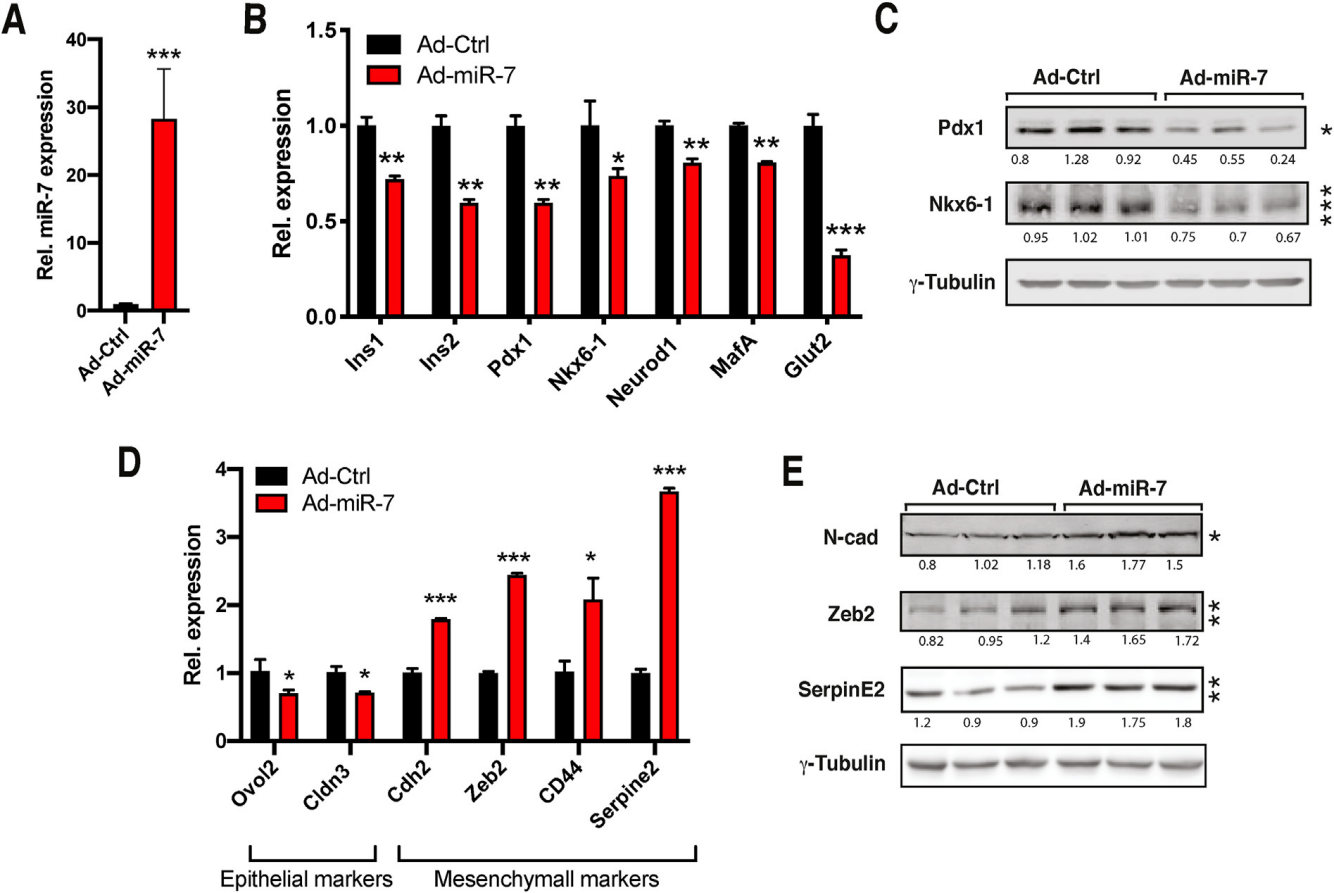
**miR-7 overexpression induces genes involved in EMT in MIN6 cells**. (A–B) Expression of miR-7 (A) and β-cell specific (B) genes in MIN6 cells infected with miR-7 (Ad-miR-7) or GFP (Ad-Ctrl) encoding adenovirus (n = 3). (C) Western blotting for β-TFs *Pdx1* and *Nkx6-1* and loading control γ-tubulin (n = 3). (D) mRNA levels of epithelial and mesenchymal markers in Ad-miR-7-infected MIN6 cells (n = 3) (E) Western blotting for indicated mesenchymal markers. Unpaired Student's t-test. Data are means ± SEM, ∗p < 0.05, ∗∗p < 0.01, and ∗∗∗p < 0.001.

To investigate the molecular mechanism underlying induction of EMT signalling in dedifferentiated β-cells, we established an *in vitro* model based on the overexpression of miR-7 in MIN6 β-cells. As expected, infection of MIN6 cells with miR-7-encoding adenovirus triggered the dedifferentiation of MIN6 cells as revealed by the downregulation of β-cell-specific markers compared to Ad-control infected cells (Figure 5A–C). Moreover, Ad-miR-7-mediated β-cell dedifferentiation was associated with decreased expression of a subset of epithelial markers (Figure 5D) and increased expression of mesenchymal genes such as *Cdh2*, *Zeb2*, and *Serpine2* (Figure 5E). These observations revealed a cell-autonomous function of miR-7 in altering the identity of β-cells and inducing the expression of EMT-related genes.

### 3.6 *Pdx1* controls *Ovol2* gene expression to repress *Zeb2* expression in β-cells

**Figure 6:**
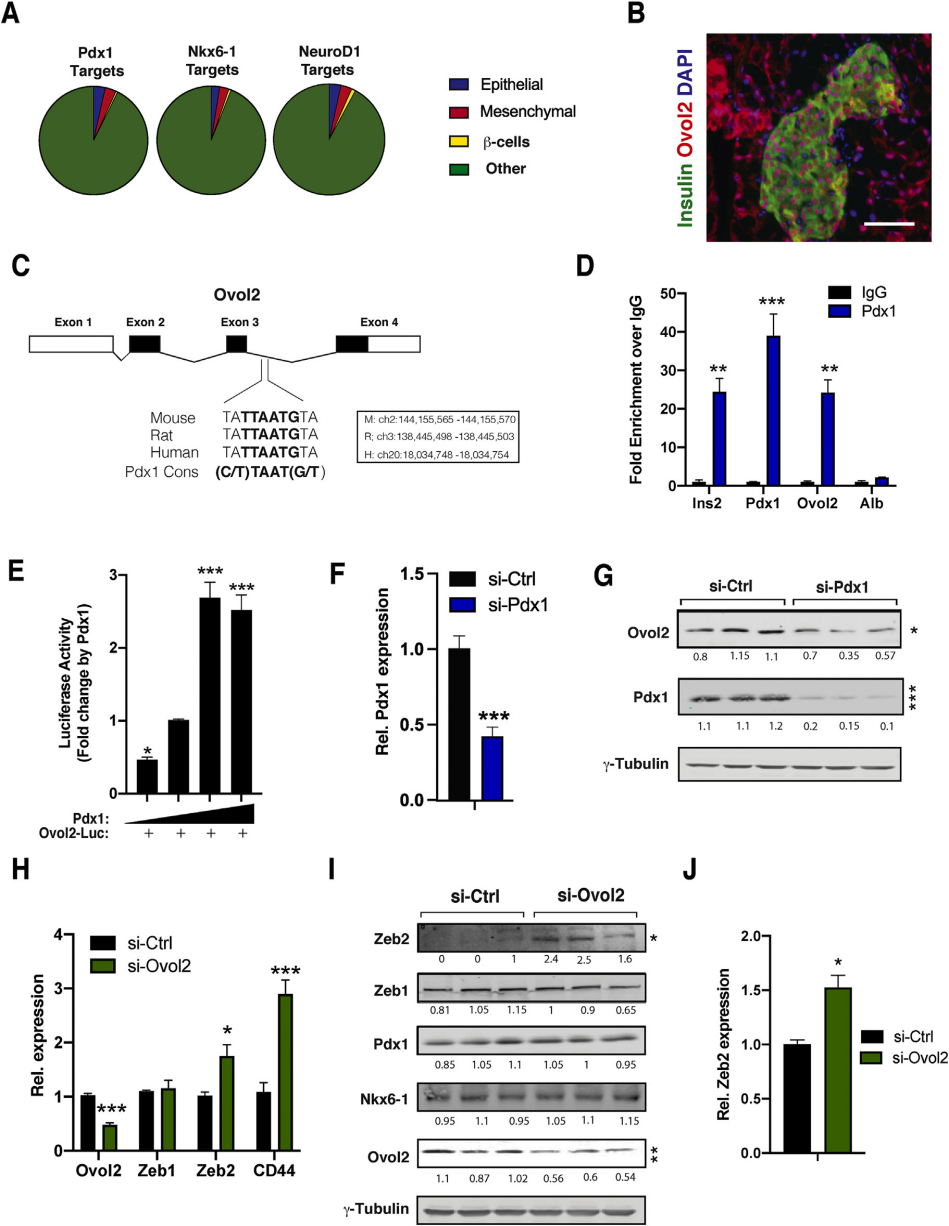
**β-TFs *Pdx1* stimulates *Ovol2* expression and triggers *Zeb2* downregulation in β-cells.** (A) Data mining of ChIP-seq data sets reveals β-TFs recruitment to epithelial and mesenchymal genes in adult mouse islets. ChIP data sets from Tenant et al., 2013 (B) Representative co-immunostaining of insulin (green) and *Ovol2* (red) in mouse pancreatic sections. Nuclei (blue) revealed by DAPI staining. Scale bar: 40 μm. (C) An evolutionarily conserved *Pdx1*-binding site located in *Ovol2* intron 3. (D) Recruitment of *Pdx1* to the *Ovol2* gene by CUT&Tag (n = 3). (E) Relative luciferase levels of a reporter construct harbouring the intronic region of *Ovol2* containing the *Pdx1* site in HEK293 cells co-transfected with *Pdx1* (100, 200, 400, and 800 ng) (n = 3). (F) *Pdx1* RNAi reduces *Ovol2* mRNA (n = 3). (G) Western blotting analysis of lysates from *Pdx1* RNAi in MIN6 cells. (H) Expression of indicated epithelial and mesenchymal genes in MIN6 depleted of *Ovol2* by RNAi (n = 3). (I) Western blotting analysis of lysates from MIN6 cells depleted of *Ovol2* by RNAi using antibodies against the indicated proteins. (J) Expression of *Zeb2* in EndoC-bH1 cells depleted of *Ovol2* by RNAi (n = 3). Unpaired Student's t-test. Data are means ± SEM. ∗p < 0.05, ∗∗p < 0.01, and ∗∗∗p < 0.001.

Because mature β-cells retain the expression of several epithelial markers, we hypothesised that β-TFs are required to maintain the epithelial gene programme within β-cells to prevent their reprogramming by EMT. To test this, we examined publicly available adult mouse islet ChIP-seq data sets for *Pdx1*, *Nkx6-1*, and *NeuroD1* [55,56]. This analysis revealed that these β-TFs were recruited to β-cell-specific loci and also bound regulatory elements found in a large number of epithelial-specific genes expressed in islet endocrine cells (Figure 6A and Supplementary Table 6). We estimated that *Pdx1*, *Nkx6-1*, and *NeuroD1* were recruited to 10.6 ± 1.5% of genes with a biological function specific to epithelial cells.

Given that *Pdx1* is the most downregulated β-TFs in Tg7 islets, we investigated its role in modulating epithelial and mesenchymal gene expression signalling in dedifferentiated β-cells. Among epithelial-specific genes downregulated in dedifferentiated β-cells of Tg7 and *db/db* islets was *Ovol2*, a fundamental regulator of epithelial cell differentiation and plasticity in several organs [[57], [58], [59]]. *Ovol2* encodes a transcriptional repressor preserving epithelial cell identity by suppressing a wide array of EMT genes [57,58]. However, its role in pancreatic β-cells remains completely unknown. Immunostaining on mouse pancreas sections revealed that *Ovol2* was expressed in β-cells (Figure 6B). Interestingly, we identified an evolutionarily conserved *Pdx1*-binding site (C/T)TAAT(G/T) within intron 3 of the *Ovol2* locus (Figure 6C), suggestive of a functional interaction between *Pdx1* and the *Ovol2* gene in β-cells. Using CUT&Tag, an assay enabling the identification of transcription factor target genes in unfixed cells [49], we determined whether *Pdx1* is directly recruited to this intronic sequence within the *Ovol2* gene. Our results indicated that *Pdx1* binds to its binding site on the *Ovol2* locus as well as to previously reported regulatory elements in the *Ins2* gene and its own promoter (Figure 6D). Furthermore, the overexpression of *Pdx1* resulted in a dose-dependent increase in the activity of a luciferase reporter bearing that specific *Ovol2* intronic regulatory sequence (Figure 6E). RNA interference (RNAi)-mediated inactivation of *Pdx1* resulted in a 50% decrease in *Ovol2* expression (Figure 6G, H), indicating that *Pdx1* is recruited to the *Ovol2* gene and stimulates its transcription in cells.

To test whether decreased *Ovol2* expression causes induction of EMT genes in β-cells, we depleted *Ovol2* expression using RNAi in MIN6 cells. Our results revealed increased *Zeb2* and *Cd4*4 mRNA levels in *Ovol2*-depleted MIN6 (Figure 6H, I), whereas the expression of epithelial-specific genes remained essentially unchanged (Supplementary Figure 5C, D). Importantly, the depletion of *Ovol2* in the human β-cell line EndoC-bH1 also resulted in *Zeb2* induction (Figure 6J). Conversely, we did not measure any change in the expression of any β-cell-specific genes following *Ovol2* inactivation in both MIN6 and EndoC-bH1 cells (Supplementary Figure 5A–C), indicating that impaired epithelial gene expression is insufficient to trigger β-cell dedifferentiation in diabetes. In light of the direct recruitment of *Ovol2* to the *Zeb2* gene in different cell types [57,58], our results suggested that *Ovol2* is required to transcriptionally suppress *Zeb2* expression in mature β-cells and prevent their mesenchymal reprogramming.

### 3.7 EMT genes are upregulated in islets of individuals with T2D

**Figure 7:**
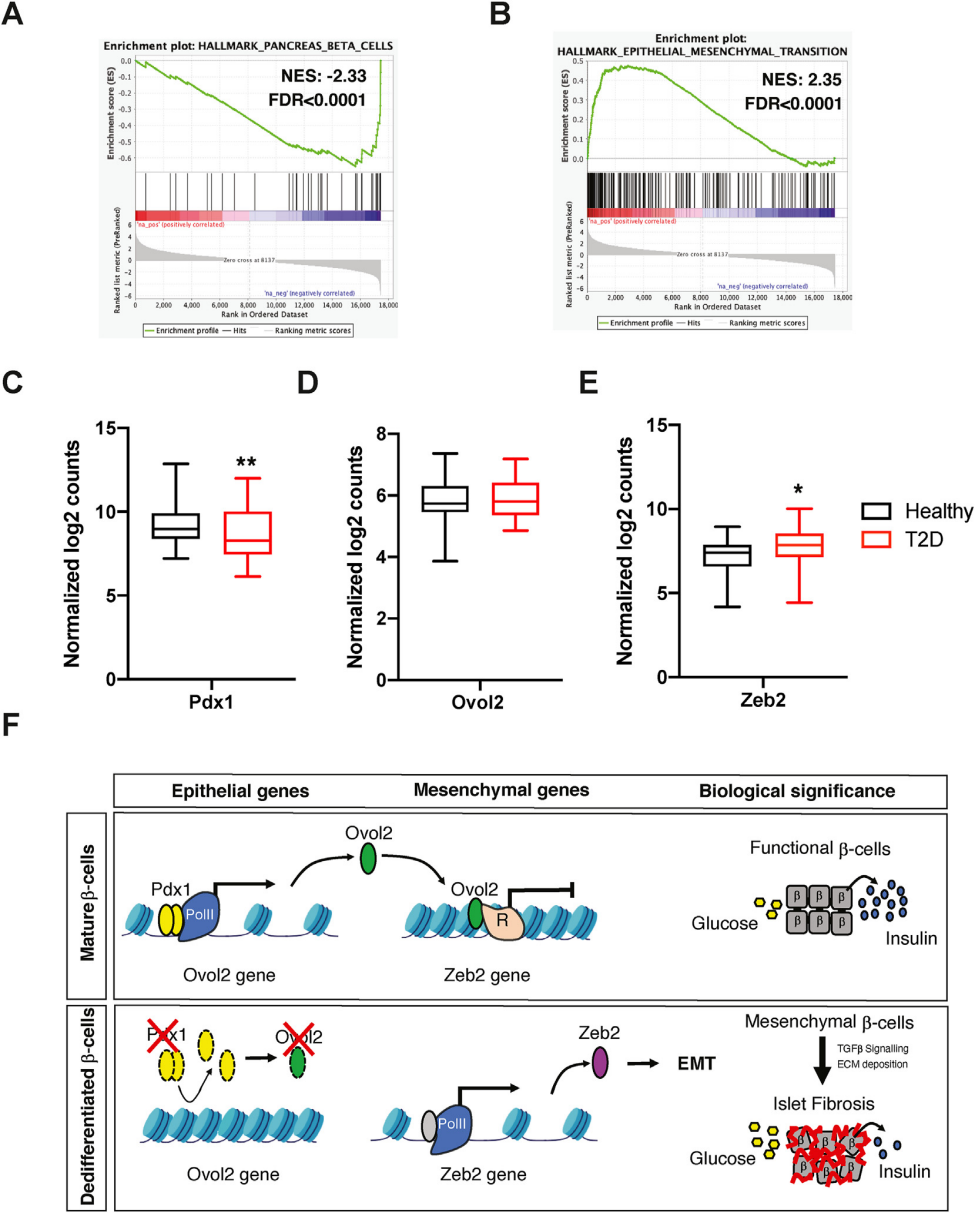
**Significance of the *Pdx1*/*Ovol2*/*Zeb2* axis in islets of individuals with T2D**. (A–B) Enrichment plot of the pancreatic beta cells (A) and epithelial to mesenchymal transition (B) hallmark genes in islets from T2D subjects. (C–E) Expression of *Pdx1* (C), *Ovol2* (D), and *Zeb2* (E) in T2D and healthy subjects. (F) Proposed model: The differentiation state of mature β-cells is defined by the activity of β-TFs such as *Pdx1*, which transactivates genes maintaining β-cell identity and function. In the current study, we show that *Pdx1* preserves the identity of β-cells by stimulating the expression of a subset of epithelial-specific genes suppressing EMT-related genes (*Ovol2*), thereby preventing mesenchymal reprogramming of β-cells. Downregulation of *Pdx1* expression in dedifferentiated β-cells decreases its occupancy on β-cell-specific and epithelial genes, thereby leading to the induction of EMT genes (*Zeb2*) and islet fibrosis.

We determined the pathological significance of the *Pdx1/Ovol2/Zeb2* axis in T2D subjects by analysing a recently published RNA-seq dataset generated from islets isolated from 58 healthy and 28 T2D subjects [43]. As expected, we found an overrepresentation of genes associated with β-cell function (*Pdx1*, *Nkx6-1*, *Pax4*, and *Slc2a2*) that was significantly downregulated in T2D individuals compared to healthy subjects (Figure 7A,C), suggestive of compromised β-cell identity. Interestingly, this correlated with an upregulation of genes associated with EMT signalling (Figure 7B). Although we did not observe changes in *Ovol2*, we found significantly higher *Zeb2* expression in T2D (Figure 7C, D), largely corroborating our *in vivo* and *in vitro* findings. The fact that *Ovol2* expression was unaffected suggested that genetic diversity and/or medication may have normalised *Ovol2* expression in these T2D patients. Nevertheless, our study highlighted how alteration of β-cell identity impacts the islet microenvironment by modulating genes associated with EMT and fibrosis (Figure 7E)

## 4 Discussion

Changes in the integrity of epithelial cells trigger their reprogramming into mesenchymal cells through the induction of EMT [60]. Conversely, EMT signalling is also activated following the dedifferentiation of non-epithelial cells and correlates with augmented progenitor and stem cell-like properties [61]. Previous research indicated that pancreatic β-cells from patients with T2D are labelled with mesenchymal markers [28], but the pathological significance and underlying mechanisms remain to be elucidated. Herein, we report that EMT signalling is prompted following β-cell dedifferentiation. Using mice overexpressing miR-7 as a model of β-cell dedifferentiation, we found that genes associated with EMT signalling were already upregulated in dedifferentiated β-cells from prediabetic mice, suggesting that EMT represents a cell-autonomous effect caused by loss of β-cell identity rather than a secondary effect of hyperglycaemia. However, EMT gene expression appeared to be somewhat augmented in the 12w Tg7 islets, suggesting that hyperglycaemia and/or other circulating factors may potentiate EMT signalling in diabetes. This EMT programme correlates with a weakening of islet cell:cell interactions, remodelling the ECM and fibrosis, a process reminiscent of a dysregulated response to tissue injury [62]. At the molecular level, we revealed that loss of β-cell identity linked to loss of β-cell TF expression resulted in decreased expression of epithelial-specific genes and a corresponding increase in the expression of genes involved in EMT, TGFβ signalling, and collagen deposition. We could ascertain that EMT is an intrinsic feature of islets from *db/db* mice and T2D subjects, underscoring the pathological relevance of EMT in diabetes.

While many β-TFs are required to maintain β-cell identity by transactivating genes involved in glucose transport, signalling, metabolism, and membrane potential underlying GSIS, we demonstrated herein that β-TFs control an epithelial gene programme preventing the mesenchymal reprogramming of β-cells. We provide evidence that β-TFs *Pdx1* transactivates *Ovol2,* encoding an epithelial-specific regulator and suppressor of EMT. Loss of *Pdx1* decreases *Ovol2* levels and consequently triggers mesenchymal reprogramming of β-cells through the de-repression of EMT-TF *Zeb2*. These observations agree with reports indicating that loss of *Ovol2* expression is sufficient to trigger EMT reprogramming [[63], [64], [65]] and that *Zeb2* causes mesenchymal reprogramming of different types of cells and contributes to organ fibrosis [66,67]. Furthermore, other β-TFs such as NKX6-1 and NEUROD1 are also recruited to the *Ovol2* loci in mouse islets [56]. Interestingly, ChIP experiments revealed that β-TFs also bind multiple genes encoding key regulators in mesenchymal cell lineages [68,69]. This raises the possibility that direct transcriptional repression of EMT genes by β-TFs also contributes to locking the identity of mature β-cells. Importantly, we observed that the depletion of *Ovol2* in MIN6 and EndoC-bH1 does not alter the expression β- of β-TFs or any other genes preserving the functional identity of β-cells, indicating that dysregulation of the epithelial gene programme within β-cells does not contribute to their dedifferentiation in diabetes. Our research showed a high-ordered regulatory pathway whereby changes in the expression of EMT genes in β-cells occur secondary to β-cell dedifferentiation in diabetes.

Among the repertoire of β-cell regulators controlling insulin gene transcription and GSIS, only *Pax6* and *Gata6* transcripts possess miR-7 binding sites in their 3-UTR. We and others have demonstrated that miR-7 binds and represses the expression of *Pax6* [24,25,70] and *Gata6* in β-cells [22]. Genetic deletion of either *Pax6* or *Gata6* results in hyperglycaemia and decreases the expression of several β-TFs and regulators of GSIS [14,15,71]. These observations indicate that miR-7-mediated dedifferentiation is in part elicited by the downregulation of *Pax6* and *Gata6* expression in diabetes. Interestingly, *Pax6* and *Gata6* bind to highly conserved sequences within enhancer elements found upstream of the *Pdx1* gene [15,[71], [72], [73], [74]]. Hence, this strongly suggests that the repression of *Pax6* and *Gata6* by miR-7 impedes the transactivation of the *Pdx1* gene and prevents activation of *Ovol2* transcription, thereby inducing EMT-related gene expression in dedifferentiated β-cells.

Although there is compelling evidence for EMT in regulating organ fibrosis and cancer, EMT also plays a fundamental role in wound healing, tissue repair, and regeneration [26]. In many of these latter cases, TGFβ signalling triggers an EMT process promoting recruitment of inflammatory cells that modulate cell:cell interactions, motility, and ECM deposition, but also impacts local angiogenesis, allowing sufficient nutrients and oxygen to support tissue repair [75]. Although islets from the Tg7 mice showed enlargement of the intercellular space between cells as revealed by a loss of islet cell:cell contacts, our initial analyses did not reveal any dissemination of *tdTomato*^+^ dedifferentiated β-cells into the exocrine pancreas or any other organs (data not shown). This suggests changes in islet cell:cell contacts do not provide any migratory advantages to β-cells as seen in cancer and metastasis. However, given that the dissociation of mouse and human islets is known to induce EMT *in vitro* [76], β-cell dedifferentiation may weaken islet cell:cell contacts and contribute in part to the mesenchymal reprogramming of β-cells. Interestingly, islets from diabetic subjects present increased vasculature density caused by the dilation of pre-existing islet blood vessels, which is thought to enable the adaptation of pancreatic islets to insulin requirements [77,78]. Our results revealed a strong correlation between EMT and the induction of genes regulating vasculature plasticity in both the normoglycemic and diabetic Tg7 mice, thus suggesting that blood vessel thickening may be modulated by vasoregulatory factors secreted in a paracrine manner by mesenchymally reprogrammed β-cells. One candidate factor that could influence both vasculature plasticity and remodelling of the ECM is *Serpine2*, which was previously reported to regulate coagulation and angiogenesis [79,80], and TGFβ-induced ECM deposition and fibrosis [[81], [82], [83]]. Given that no significant changes in the proportions of islet myofibroblastic stellate cells were observed in our Tg7 sc-RNA-seq dataset, this suggests an active role of dedifferentiated β-cells in regulating islet vasculature remodelling, ECM deposition, and islet fibrosis.

Genetic lineage studies in the pancreas indicated that reprogramming of the β-cell identity in response to metabolic stress appears to underlie a pathological and maladaptive mechanism in diabetes rather than a regeneration process leading to recovery of β-cell mass. Therefore, it is thus possible that the mesenchymal reprogramming of β-cells we report herein may increase islet cell plasticity and promote β-cell conversion into other cell endocrine types. Intriguingly, it has been shown that both *Ovol2* and *Zeb2* modulate cellular plasticity by fine-tuning the hybrid epithelial/mesenchymal states of *Cd44*^+^ cancer cells [84,85]. However, given that EMT is a multistage process [84,[86], [87], [88]], it is also possible that this EMT process represents a snapshot of a long-term regenerative process aimed at restoring β-cell mass. Indeed, in extreme diabetic mouse models of β-cell depletion, regeneration of insulin-producing cells is controlled by TGFβ signalling [89], whereas acute β-cell depletion by streptozotocin triggers the repopulation of β-cell mass from a rare population of VIM^+^/MAFB^+^ cells [90]. Further research is required to understand the extent to which EMT signalling affects the plasticity and regeneration of β-cells and other islet cell types.

In summary, our study shows that β-cell dedifferentiation induced by miR-7 triggers the expression of genes associated with EMT and a chronic response to tissue injury. In light of the reversibility of EMT, our research suggests that medical intervention fostering the epithelial phenotype of β-cells and/or preventing their mesenchymal reprogramming may be used to improve glycaemic controls in diabetes.

## Author contribution

D.S. de J., T.M., Y.v.O., Y.B., P.C., and M.L. designed all of the experiments. D.S. de J, T.M., Y.v.O., Y.B., P.E., P.C., A.T., and M.L. performed the experiments. D.S. de J., T.M., Y.v.O., Y.F.W., Y.B., E.K., P.E., P.C., W.D., V.S., and M.L. analysed the data. M.S. provided the Tg7 mouse line. P.M., A.M.J.S., and G.A.R. coordinated the procurement and culture of human islet samples. D.S. de J., T.M., Y.v.O., P.C., and M.L. prepared figures. G.A.R wrote part of the manuscript. M.L. directed the study and wrote the manuscript. All the authors read and approved the final version of the manuscript.

## Figures and Tables

**figs1:**
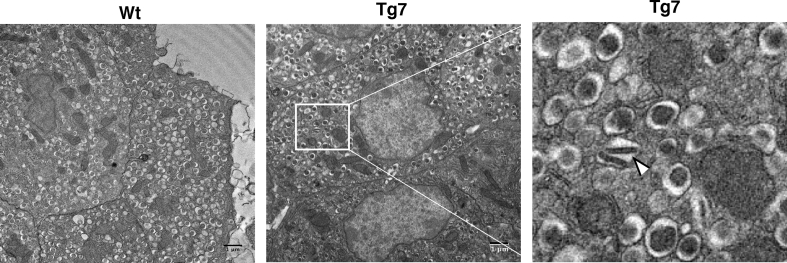


**figs2:**
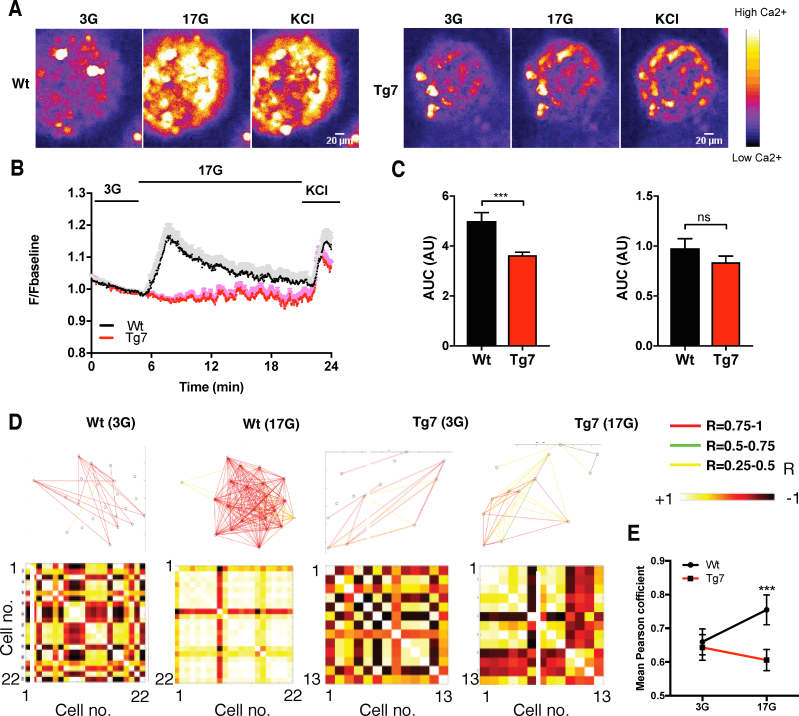


**figs3:**
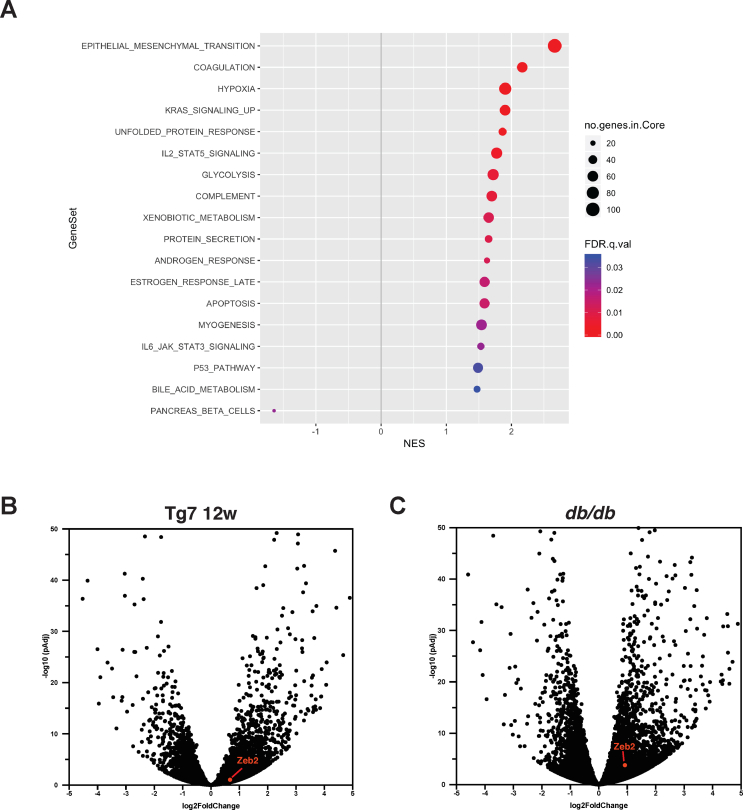


**figs4:**
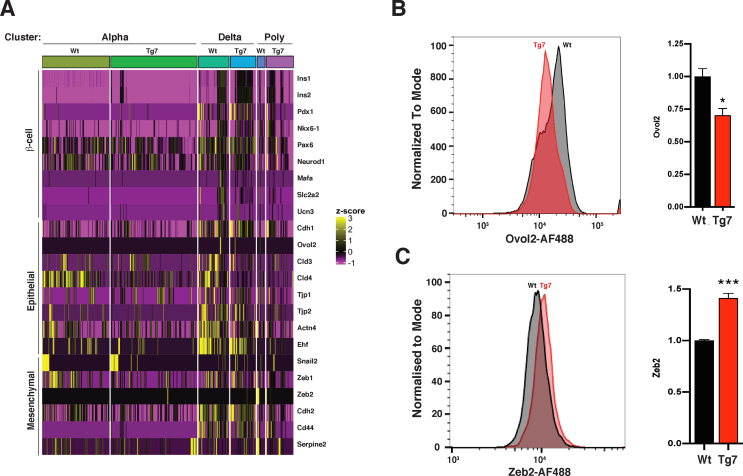


**figs5:**